# Supplementary material for: Efficacy of Remdesivir-Containing Therapy in Hospitalized COVID-19 Patients: A Prospective Clinical Experience
Source: J Clin Med. 2021 Aug 24;10(17):3784. doi: 10.3390/jcm10173784 (PMC8432083; doi:10.3390/jcm10173784)
Supplement: Supplementary file 1 [file jcm-10-03784-s001.zip › jcm-1309588-supplementary.pdf]

Supplementary Table S1. Univariate analysis before propensity score matching.

Variables	No remdesivir N = 113 (%)	Remdesivir N = 294 (%)	*P* value
Male sex	70 (62%)	250 (80%)	0.760
Age, year, mean (±SD)	62.5 (±20)	63.2 (± 15.3)	0.717
Days from symptoms/positive nasopharyngeal swab to admission	22 (23%)	7 (7%)	0.573
Comorbidities	77 (68%)	213 (70%)	0.919
Cardiovascular disease	17 (15%)	33 (11%)	0.203
COPD	19 (17%)	31 (10%)	0.051
Chronic kidney disease	11 (12%)	18 (6%)	0.256
Liver cirrhosis	2 (2%)	12 (4%)	0.293
Diabetes mellitus	21 (19%)	53 (17%)	0.293
Solid lung cancer (primary or metastasis)	1 (1%)	3 (1%)	1.000
Fever	56 (50%)	246(79%)	**<0.001**
Cough	33 (29%)	157 (50%)	**<0.001**
Dyspnea	42 (37%)	178 (57%)	**<0.001**
Gastrointestinal symptoms (diarrhea, abdominal discomfort, nausea, vomiting)	16 (14%)	58 (18%)	0.271
Fatigue	21 (19%)	57 (19%)	1.000
Pharyngodynia	6 (5%)	14 (5%)	1.000
Rhinitis	3 (3%)	15 (5%)	0.329
Arthralgia/Myalgia	13 (12%)	45 (14%)	0.460
Anosmia	3 (3%)	9 (3%)	1.000
Conjunctivitis	0 (0%)	2 (1%)	0.647
Chest pain	5 (4%)	11 (3%)	0.655
Signs of overload (limb edema and/or pulmonary stasis)	9 (9%)	2 (1%)	**<0.001**
Parenchymal thickening	72 (64%)	232(74%)	**0.046**
Interstitial lung disease	16 (14%)	15 (5%)	**<0.001**
Pleural effusion	21 (18%)	26 (9%)	**0.012**
Cardiomegaly	8 (7%)	16 (5%)	0.438

Variables	No remdesivir<br>N = 113 (%)	Remdesivir<br>N = 294 (%)	*P* value
Bronchiectasis/ Emphysema	27 (24%)	50 (15%)	**0.032**
White Blood cells x10^3/uL, mean (±SD)	7.38 (± 3.59)	8.06 (± 5.99)	0.287
Neutrophils x10^3/uL, mean (±SD)	5.6 (± 3.61)	6.31 (± 5.31)	0.211
Platelets x10^3/uL, mean (±SD)	247.66 (± 100.85)	218.47 (± 81.73)	**0.004**
D-dimer ng/mL, mean (±SD)	1365.71 (± 1456.18)	814.91 (± 766.45)	**<0.001**
Ferritin ng/mL, mean (±SD)	692.35 (± 942.17)	645.02 (± 489.52)	0.591
Procalcitonin ng/mL, mean (±SD)	1.2 (± 6.4)	0.61 (± 3.73)	0.334
LDH mU/mL, mean (±SD)	286.46 (± 163.73)	302.37 (± 119.57)	0.300
CPK U/L, mean (±SD)	278.9 (± 1430.6)	149.47 (± 161.35)	0.170
Lactates mmol/L, mean (±SD)	1.57 (± 0.36)	1.33 (± 0.83)	0.328
C-reactive protein mg/dL, mean (±SD)	4.91 (± 6.61)	8.23 (± 21.13)	0.116
PaO_2/FiO_2, mean (±SD)	315.67 (± 117.14)	329.94 (± 98.36)	0.411
Aspartate Transaminase U/L, mean (±SD)	34.16 (± 47.76)	35.24 (± 27.41)	0.784
Alanine transaminase U/L, mean (±SD)	32.2 (± 28.42)	33.24 (± 28.84)	0.755
Bacterial co-infection	24 (21%)	65 (20%)	0.928
Days of hospitalization, mean (±SD)	16.1 (± 17.62)	15.0 (± 9.98)	0.487
Days to nasopharyngeal swab negativization, mean (±SD)	24.77 (± 17.1)	22.6 (± 13.77)	0.387
30-day mortality	4 (4%)	17 (6%)	0.411
No Steroids	92 (81%)	289 (93%)	**<0.001**
Antibiotics (excluding Macrolides)	65 (58%)	83 (27%)	**<0.001**
Macrolides	74 (65%)	146 (46%)	**0.001**
No Low-molecular-weight heparin	73 (65%)	76 (87%)	0.749
Low-flow oxygen therapy/no need of oxygen therapy	89 (79%)	275 (79%)	**0.044**
HFNC/NIV	17 (15%)	33 (10%)	0.155
Mechanical ventilation	1 (1%)	10 (3%)	0.200

Legend. COPD: chronic obstructive pulmonary disease; LDH: lactate dehydrogenase; CPK: creatine phosphokinase; HFNC: high flow nasal cannula; NIV: non-invasive ventilation.

Supplementary Table S2. Univariate analysis after propensity score matching.

Variables	No remdesivir<br>N = 71 (%)	Remdesivir<br>N = 71 (%)	*P* value
Male sex	48 (68%)	52 (73%)	0.466
Age, year, mean (±SD)	63 (±19)	74 (± 12)	**<0.001**
Days from symptoms/positive nasopharyngeal swab to admission	20 (28%)	6 (8%)	0.279
Comorbidities	46 (65%)	63 (89%)	**<0.001**
Cardiovascular disease	14 (20%)	20 (28%)	0.265
COPD	12 (17%)	17 (24%)	0.323
Chronic kidney disease	7 (10%)	8 (11%)	0.815
Liver cirrhosis	0 (0%)	5 (7%)	0.104
Diabetes mellitus	7 (10%)	18 (25%)	**0.016**
Solid lung cancer (primary or metastasis)	1 (1%)	3 (4%)	0.455
Fever	33 (46%)	49 (69%)	**0.008**
Cough	17 (24%)	37 (52%)	**<0.001**
Dyspnea	34 (48%)	40 (56%)	0.277
Gastrointestinal symptoms (diarrhea, abdominal discomfort, nausea, vomiting)	7 (10%)	16 (23%)	**0.035**
Fatigue	14 (20%)	7 (10%)	0.115
Pharyngodynia	2 (3%)	2 (3%)	1.000
Rhinitis	8 (11%)	4 (6%)	0.295
Arthralgia/Myalgia	6 (8%)	6 (8%)	1.000
Anosmia	1 (1%)	2 (3%)	0.801
Conjunctivitis	0 (0%)	2 (3%)	0.160
Chest pain	3 (4%)	3 (4%)	1.000
Signs of overload (limb edema and/or pulmonary stasis)	6 (8%)	0 (0%)	**0.012**
Parenchymal thickening	38 (54%)	50 (70%)	**0.038**
Interstitial lung disease	9 (13%)	4 (6%)	**<0.001**
Pleural effusion	18 (25%)	7 (10%)	0.118
Cardiomegaly	6 (8%)	3 (4%)	0.417
Bronchiectasis/ Emphysema	18 (25%)	22 (31%)	0.724
White Blood cells x10^3/uL, mean (±SD)	7.92 (± 4.54)	9.9(± 9.15)	0.107
Neutrophils x10^3/uL, mean (±SD)	6.45 (± 4.60)	8.56 (± 8.77)	0.075
Platelets x10^3/uL, mean (±SD)	240.48 (± 90.1)	219.37 (± 89.0)	0.165
D-dimer ng/mL, mean (±SD)	1337.99 (± 1349.44)	1065.42 (± 902.01)	0.160
Ferritin ng/mL, mean (±SD)	669.77 (± 723.69)	704.94 (±407.35)	0.722
Procalcitonin ng/mL, mean (±SD)	0.64 (± 1.34)	1.58 (± 5.99)	0.204
LDH mU/mL, mean (±SD)	339.46 (± 212.05)	326.13 (± 116.49)	0.644
CPK U/L, mean (±SD)	397.77 (±1716.54)	141.90 (± 125.43)	0.214

**Variables**	**No remdesivir**<br>**N = 71 (%)**	**Remdesivir**<br>**N = 71 (%)**	***P* value**
Lactates mmol/L, mean (±SD)	1.5 (± 0.14)	1.51 (± 0.74)	0.874
C-reactive protein mg/dL, mean (±SD)	6.3 (± 8.77)	8.45 (± 6.77)	0.103
PaO_2/FiO_2, mean (±SD)	308.32 (± 80.86)	303.05 (±99.85)	0.731
Aspartate Transaminase U/L, mean (±SD)	44.79 (± 63.35)	32.62 (± 15.04)	0.119
Alanine transaminase U/L, mean (±SD)	34.8 (± 29.4)	27.71 (± 15.39)	0.075
Bacterial co-infection	12 (17%)	17 (24%)	0.258
Days of hospitalization, mean (±SD)	16.4 (± 17.1)	17.8 (± 12.3)	0.591
Days to nasopharyngeal swab negativization, mean (±SD)	23.2 (± 9.5)	23 (±10)	0.511
30-day mortality	2 (3%)	7 (10%)	0.515
No Steroids	59 (83%)	55 (77%)	0.926
Antibiotics (excluding Macrolides)	34 (48%)	25 (35%)	0.345
Macrolides	45 (63%)	41 (58%)	0.953
No Low-molecular-weight heparin	57 (80%)	55 (77%)	0.749
Low-flow oxygen therapy/no need of oxygen therapy	53 (75%)	52 (73%)	0.760
HFNC/NIV	17 (24%)	17 (24%)	0.877
Mechanical ventilation	2 (3%)	3 (3%)	0.848
Propensity score, mean (±SD)	0.58 (± 0.31)	0.57 (± 0.32)	0.895
Propensity score weight simple size	71 (100%)	71 (100%)	1.000

Legend. COPD: chronic obstructive pulmonary disease; LDH: lactate dehydrogenase; CPK: creatine phosphokinase; HFNC: high flow nasal cannula; NIV: non-invasive ventilation.

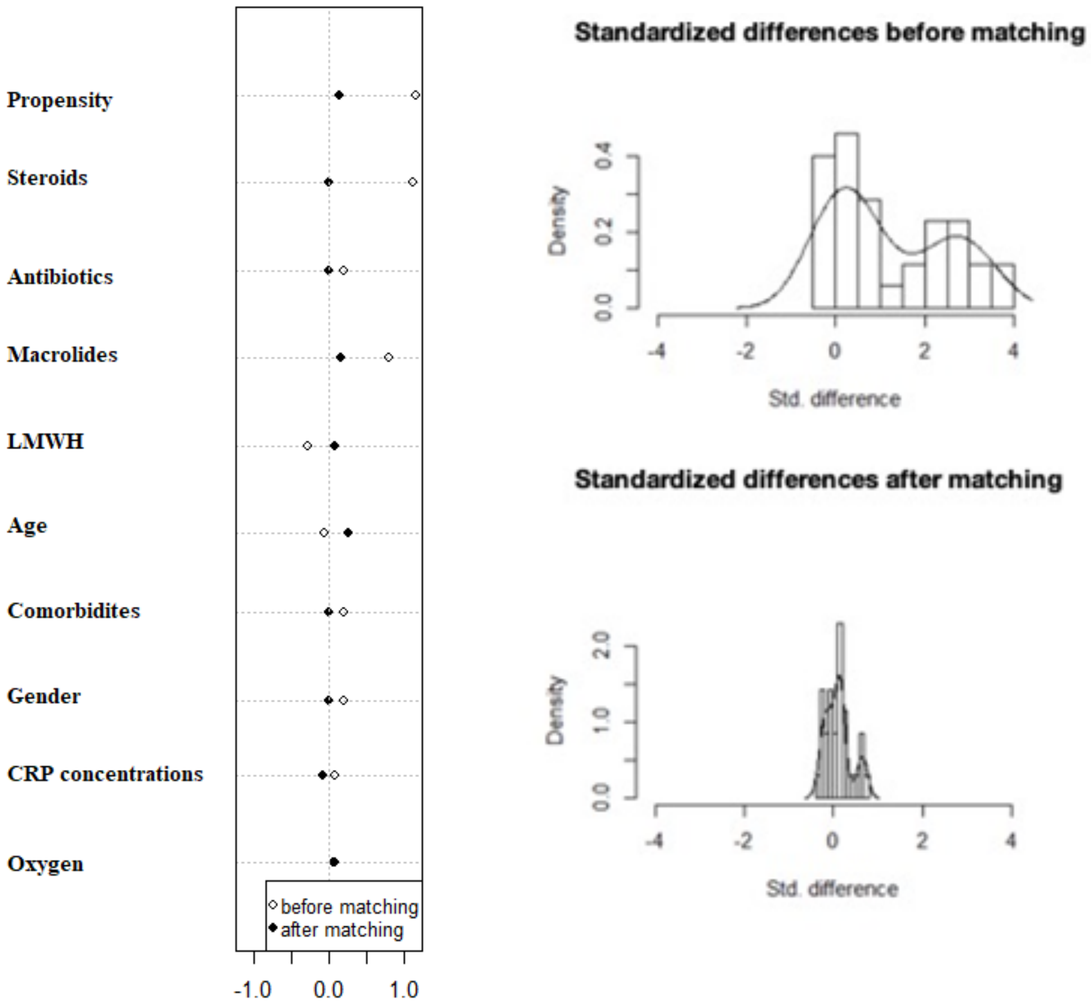

Supplementary Figure S1. Standardized differences before and after propensity score matching.
